# Construction of an integrated sports-medical-older adults care service system for early health intervention in rural Shandong

**DOI:** 10.3389/fpubh.2026.1958909

**Published:** 2026-11-09

**Authors:** Qiang Chen

**Affiliations:** Department of Public Basic Courses, Shandong University of Art and Design, Jinan, Shandong, China

**Keywords:** China’s national health strategy, collaborative governance, integrated sports, medical care and care for older adults, primary preventive health intervention, rural aging

## Abstract

With the implementation of the “Healthy China” strategy and the ongoing deepening of population aging, preventive public health services covering the entire life cycle have become a key national development priority. Shandong Province faces severe pressures from rural aging: constrained by rural economic conditions and geographical factors, health and wellness resources are unevenly distributed, and the integration of sports, medical care, and care for older adults services remains highly fragmented. Most existing studies focus on urban settings and emphasize passive care for older adults following the onset of illness; few are oriented toward early health intervention or aim to establish a multi-stakeholder collaborative system tailored to the realities of rural Shandong. Consequently, there is a significant research gap in the field of localized governance of rural aging. Based on Synergy Theory, this study employs a two-stage systematic literature review method to integrate domestic academic literature, international research on aging governance, and official statistics and policy documents. It analyzes the current imbalance between supply and demand for integrated rural services, explores the intrinsic drivers and barriers to interdepartmental service coordination, and ultimately constructs a practical, integrated system of sports, medical, and care for older adults services tailored to rural older adults. The findings of this study can provide optimized policy proposals for rural health and care for older adults in Shandong Province, expand the boundaries of localized practice for Synergy Theory, and simultaneously establish a replicable practical paradigm for promoting the integration of rural public services in aging regions worldwide.

## 1 Introduction

Global public health governance is gradually shifting toward a full-life-cycle governance system, and at the same time, China is vigorously promoting proactive early health interventions. Against this backdrop, healthy aging has become a core policy priority for addressing the challenges of rural population aging worldwide. Shandong Province is one of the provinces with the highest levels of population aging in China, and rural health services for older adults face significant structural challenges: uneven resource allocation, fragmented cross-sectoral governance, and a low degree of integration among physical activity and health interventions, medical services, and long-term care. These issues severely hinder the equitable and sustainable advancement of healthy aging in rural areas. This study adopts synergy theory, a complex-system theory which explains how interdependent subsystems and stakeholders interact to generate emergent collective outcomes that exceed the effect of isolated individual actors. In this framework, early preventive health intervention is defined as the core order parameter shaping system evolution, while governmental fiscal and institutional regulation functions as the primary control parameter. This localized adaptation of synergy theory helps unpack cross-departmental coordination barriers and collaborative mechanisms within the rural sports-medical-older adults care service system.

Shandong Province bears one of China’s heaviest rural aging burdens. Official statistics show that more than 25.7% of its total population is aged 60 or above, with a large share of older adults residing in rural villages, accompanied by high prevalence of chronic metabolic diseases (1). Rural regions across Shandong exhibit substantial within-province heterogeneity: eastern counties enjoy stronger fiscal capacity and better grassroots medical resources, while western and inland rural counties face weaker public infrastructure, outmigration of young labor, and fragmented cross-sector service governance. Against this regional backdrop, exploring integrated sports-medical-older adults care becomes highly representative for understanding China’s rural aging governance. Constrained by factors such as dispersed residential patterns and insufficient fiscal funding, rural areas in China cannot simply replicate the urban model of centralized institutional elder care. The endogenous neighborhood mutual-aid model for elder care—which has emerged from rural communities based on familiar social networks—has been validated through simulations using evolutionary game theory as a practical and sustainable pathway for achieving healthy aging in rural areas (2). Internationally, Nordic countries rely on user satisfaction scoring and ranking systems to regulate the quality of older adult care services; scholars in the field of global geriatric health have also developed standardized, tiered exercise intervention programs for older adults (3, 4). Multiple domestic empirical studies have further confirmed that there are hierarchical differences in the health and wellness needs of older adults, and that physical exercise can enhance the physical and mental health of rural older adults through multiple mediating effects (5, 6). In summary, based on the aforementioned theoretical findings and practical experience, this study constructs an integrated system of sports, medical care, and older adult care services tailored to the regional characteristics of rural Shandong Province.

Numerous existing studies have explored the integration of physical activity, medical care, and long-term care services. Empirical research consistently indicates that rural older adults have a vast array of unmet public health needs, including professional fitness guidance, exercise programs tailored to older adults, and standardized preventive health management services (7). However, existing research still has three notable limitations: First, most studies focus on urban communities and concentrate on post-illness rehabilitation and convalescence, with very few studies addressing proactive, early-stage health interventions in rural settings; Second, past research has largely emphasized the optimization of individual service sectors, neglecting the multi-stakeholder coordination mechanisms among multiple departments such as sports, health, and civil affairs; third, very few studies have integrated the demographic structure and socioeconomic realities of rural Shandong to establish an integrated service system with practical feasibility. Consequently, there is a clear research gap in the areas of localized early health interventions in rural settings and the integration of cross-departmental services.

To address these research gaps, this study, grounded in Synergy Theory and focused on the realities of rural governance in Shandong Province, systematically explores an integrated service system combining physical fitness, healthcare, and care for older adults. The study employs a two-stage systematic literature review combined with policy analysis to clarify the current mismatch between supply and demand for integrated rural health services, institutional barriers, and underlying driving logic, and to construct a multi-stakeholder collaborative service framework centered on early-life health interventions across the entire life cycle and tailored to local conditions.

The findings of this study hold dual theoretical and practical value: at the theoretical level, they expand the boundaries of synergy theory’s application in local contexts, enrich the connotations of full-life-cycle health management and the “Healthy China” strategy, and refine the academic framework for rural public health governance in an aging society; at the practical level, they provide an evidence-based pathway for policy optimization to enhance equity in the health of rural older adults and ensure the sustainable operation of care services for older adults in Shandong Province. At the same time, the service model established by this study can serve as a practical, replicable template for all rural areas worldwide that are entering the aging phase to advance the integration of cross-sectoral public services.

## 2 Methods

This is a policy-oriented systematic literature review rather than a systematic review of clinical intervention studies. This study employs a standardized two-stage systematic literature review methodology to conduct theoretical deduction and policy analysis. Throughout the research process, no field research, questionnaire surveys, semi-structured interviews, or on-site observations were conducted, nor was any other primary data collection involving human subjects carried out. All analytical data were sourced from publicly released policy documents issued by the Chinese central government and the Shandong Provincial Government, peer-reviewed academic papers from China and abroad, and official statistical bulletins on population and public health published by the Shandong Provincial People’s Government.

### 2.1 Phase I: systematic literature search, screening, and organization

The study conducted a systematic literature search, supplemented by two rounds of literature screening, to establish a comprehensive literature database and lay a solid foundation for subsequent theoretical analysis. Domestic literature was primarily retrieved from China National Knowledge Infrastructure (CNKI); foreign journals and literature were collected through the Web of Science, Scopus, and Google Scholar. Search topics covered the integration of sports and medicine, and care service systems for older adults, early preventive health interventions in rural areas, governance of population aging, and policy practices related to the “Healthy China” strategy.

The initial search yielded over 200 journal articles and more than ten academic monographs. Following standardized screening, the literature meeting the inclusion criteria was systematically classified according to research themes, analytical perspectives, and regional research contexts. To incorporate a cross-national comparative research perspective, this study additionally incorporated international research findings from Europe, the United States, and East Asia regarding long-term care systems, preventive health management for the older adults, and interdepartmental collaborative governance of public services. Ultimately, a comprehensive literature database encompassing both Chinese and international sources was established, providing a solid foundation for the overall theoretical derivation of this study.

To ensure the reliability, standardization, and contextual relevance of the research data, this study strictly followed a two-stage systematic literature search and screening process. The literature review focused on the themes of early health intervention, the integration of physical activity, medical care, and care services for older adults in rural areas, and governance of rural aging in Shandong. By standardizing the search period, databases, keywords, and screening criteria, the study successfully identified valid literature. Specific details regarding the search and screening are presented in Table 1.

**Table 1:** Overview of literature retrieval and screening information.

Item	Details
Retrieval time frame	2010–2026
Databases	CNKI, Wanfang, Web of Science, Scopus, Google Scholar
Keywords	Early health interventions, rural aging, integration of physical fitness, medical care, and care services for older adults, collaborative governance, China’s national health strategy, rural public health services
Inclusion criteria	The included literature comprises journal articles in both Chinese and English, academic monographs from China and abroad, as well as official policy documents and authoritative statistical bulletins issued by the national government and the Shandong Provincial Government. Literature reviews must be grounded in the “Healthy China 2030” Outline, the National Fitness Plan Outline, and Shandong Province’s specialized policies on rural care services for older adults, the integration of sports and healthcare, and early health interventions for the older adults. They should focus on the themes of integrating sports, healthcare, and rural care services for older adults in rural areas; early preventive health interventions; governance of rural aging; and collaborative service provision by multiple stakeholders, and must be supported by rigorous theoretical reasoning or empirical analysis.
Exclusion criteria	Duplicate literature, conference abstracts, non-academic reports, and news commentary; studies that focus solely on urban settings or a single field (such as sports, healthcare, or rural care services for older adults) and lack an integrated perspective; and literature unrelated to early health interventions or research conducted in rural Shandong.
Literature screening process	First, an initial screening was conducted based on the titles and abstracts of the literature to exclude irrelevant studies; then, a second screening was performed through a thorough reading of the full texts to further verify the relevance of the studies, their academic quality, and their applicability to the rural context in Shandong.
Total included studies	35

To ensure the transparency and reproducibility of the retrieval and screening process, this study followed a simplified PRISMA framework, consistent with its nature as a policy-oriented systematic literature review. The initial search yielded over 200 journal articles and more than ten academic monographs. After removing duplicates, screening titles and abstracts, and assessing full texts against the pre-defined inclusion and exclusion criteria (Table 1), 35 studies were included in the final analysis.

For quality assurance, all included academic papers were assessed in terms of methodological rigor, data reliability and relevance to the rural Shandong context, and all policy documents and statistical bulletins were evaluated for authority and recency. Only documents meeting these criteria were retained.

### 2.2 Phase II: construction of the theoretical framework and the four-step progressive logical analysis method

Based on a localized and revised framework of synergetics, this study adopts this classic theory of complex systems as its core analytical perspective. The original theory of synergetics focuses on elucidating the intrinsic laws governing the self-organizing operation of open complex systems driven by order parameters and control parameters. While Western models of collaborative governance advocate for horizontal, equal negotiation among various social actors, this study differs by designating early health interventions as the core order parameter and government fiscal regulation as the primary control parameter. This approach aligns with the top-down administrative management characteristics of China’s rural areas, thereby addressing the limitation of the original theory, which cannot be directly applied to China’s grassroots governance context. Against the backdrop of the integrated development of rural care services for older adults, this study further provides a concrete interpretation and operational definition of the theory’s implications tailored to the local context. Relying on a four-step progressive logical analysis framework, the entire research process was carried out as follows:

*Inductive synthesis*: This step involves mapping the developmental trajectory and academic progress of domestic research in the field of integrated sports, healthcare, and care services for older adults; based on the compiled literature, it defines the core concepts, scope of research, and mainstream academic consensus in this field.

*Empirical analysis*: Drawing on official statistical data on population aging in Shandong Province, this step analyzes the practical challenges and structural supply–demand imbalances facing rural sports, healthcare, and care services for older adults in Shandong against the backdrop of advanced population aging.

*Comparative theoretical deduction*: Comprehensively identify all diverse stakeholders within the service system, including government functional departments (Sports Bureau, Health Commission, Civil Affairs Bureau), township medical institutions, rural facilities for older adults, community organizations, families, and third-party social service providers; elucidate the intrinsic logic of resource allocation, the criteria for the division of responsibilities and authority, and the pathways for interaction and collaboration among these stakeholders; and, based on synergy theory, establish a multidimensional collaborative operational mechanism for integrated services.

*Integration and synthesis*: Summarize all analytical conclusions and, in light of Shandong’s rural population structure, level of economic development, and the current state of public service development, formulate targeted policy optimization plans to achieve the long-term, sustainable operation of localized integrated sports, medical, and older adult care services.

This study does not merely superficially cite or apply the surface-level concepts of synergy theory; rather, it systematically expands and adapts the theory to local contexts, constructing a comprehensive theoretical application system tailored to the governance context of China’s rural aging population. Based on rigorous literature review and multidimensional logical deduction, this two-phase research design demonstrates strong academic rigor and reproducibility. This study fills an existing academic gap: previous research has rarely focused on early health intervention to specifically construct a multi-stakeholder collaborative operational mechanism for the integration of physical fitness, medical care, and care services for older adults that aligns with the regional characteristics of rural Shandong.

### 2.3 Definition of core concepts

To define the scope of the study, standardize the analytical framework, and avoid conceptual ambiguity, this study defines the core research concepts by drawing on existing academic theories. Based on the specific research context of integrated sports, medical, and care services for older adults in rural Shandong Province, the definitions of the core concepts in this study are as follows (see Table 2).

**Table 2:** Definition of core concepts in the study.

Core concept	Academic definition	Scope of application in this study
Shifting the Focus of Healthcare to Earlier Stages	A prevention-oriented health management concept that shifts the focus of health management from disease treatment to disease prevention and health promotion, covering the whole life cycle of health management	In this study, it specifically refers to the proactive health management model for rural older adults, with physical fitness and health intervention as the core
Integrated Sports, Medical, and care services for older adults	An integrated care service model for older adults that integrates resources from the three major fields of physical fitness, medical rehabilitation, and care services for older adults, achieving functional complementarity and service coordination	In this study, it specifically refers to the integrated service system built for rural older adults in Shandong Province, which is organized into three primary categories—sports intervention, medical care and care services for older adults—with nursing, rehabilitation and wellness (health preservation) provided as subordinate service modules under these primary categories
Rural Rural Care Services for Older Adults	A general term for socialized care services for older adults targeting rural older adults, covering daily care, health nursing, spiritual comfort and other contents	In this study, it specifically refers to the care services for older adults supply system in rural Shandong, based on home care, supported by community care, and supplemented by institutional care
Multi-stakeholder Collaborative Supply	A public service supply model in which multiple stakeholders including the government, enterprises, social organizations, communities and families participate together and cooperate in division of labor	In this study, it specifically refers to the functional integration, complementary advantages and coordinated operation mechanism of various stakeholders in the integrated sports-medical-older adults service system

Having systematically defined all core concepts of this study in the preceding section, this research—guided by the theory of synergy—constructs a comprehensive, closed-loop operational framework for the integrated sports, medical, and care services for older adults service system in rural Shandong Province. Centered on early preventive health interventions, this framework clarifies the key stakeholders, supporting mechanisms, specific service components, and ultimate development goals, thereby establishing an operational system capable of dynamic, cyclical optimization. The hierarchical logical structure of the entire system is illustrated in Figure 1.

**Figure 1:**
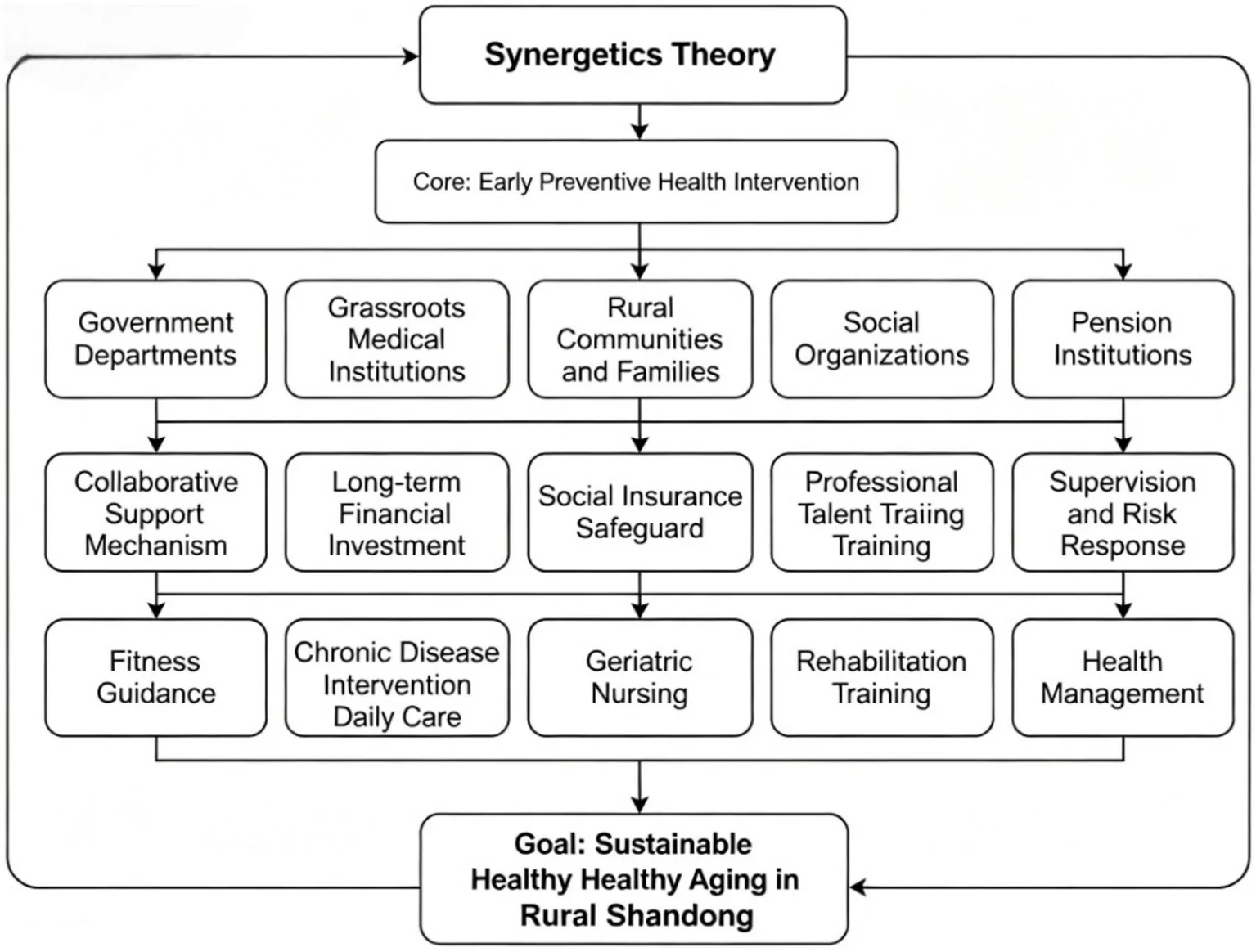
Synergetic closed-loop framework of the rural integrated sports-medical-older adults service system in Shandong Province.

## 3 Results

### 3.1 Theoretical analysis of rural integrated sports-medical-older adults care service supply in Shandong under early health intervention strategy

#### 3.1.1 Evolution context of integrated sports-medical-older adults care research

Faced with increasingly severe population aging pressures, domestic academic research on aging health services has gradually shifted from simple medical-older adults care docking to multi-dimensional fusion of sports, medical treatment and care services for older adults. Sports academia has formed abundant research results on promoting national fitness through sports-medical integration models (8). After “national fitness” and “shifting health management to early prevention” were elevated to national core strategies, the integrated development of sports and medical care has become a sustained research hotspot (9).

Taking early preventive health intervention as the core perspective, this paper sorts the theoretical connotation, internal driving forces and operational logic of rural integrated sports-medical-older adults systems, laying a standardized theoretical foundation for subsequent dilemma analysis and mechanism construction.

#### 3.1.2 Theoretical value of building a rural integrated service system in Shandong

Rural Shandong faces prominent structural contradictions between growing diversified older adults health demand and insufficient, unbalanced public service supply. The mismatch between supply capacity and service quality directly restricts the realization of healthy aging for rural residents. Theoretical research on integrated sports-medical-older adults care can clarify how public-led multi-subject collaborative supply optimizes resource allocation, and provide theoretical support for solving bottlenecks in rural care services for older adults in Shandong.

Improving the health of rural older adults and quality of life requires optimizing public service supply modes, innovating cross-department coordination mechanisms and standardizing service operation standards. The integrated sports-medical-older adults care model effectively integrates fitness guidance, chronic disease medical intervention and daily aging care, forming a full-cycle preventive health service system matching the characteristics of rural older adults.

#### 3.1.3 Driving forces for integrated service system construction

The construction of rural integrated sports-medical-older adult care services relies on complementary functional cooperation among the government, market social organizations, village communities and families to realize coordinated supply of aging health public goods (10). Moderately inclusive social care services for older adults constitute the core development goal of collaborative supply.

Under the guidance of the Healthy China strategy and whole-life-cycle health concept, institutional docking between the new rural cooperative medical scheme and rural pension insurance can be promoted at the policy level, building an open, interactive and highly coordinated multi-subject service supply operation mode.

#### 3.1.4 Core collaborative operation mechanisms of the integrated service system

##### 3.1.4.1 Multi-stakeholder collaborative support mechanism

The core of rural integrated service construction lies in guiding social forces to participate in public aging services, forming a joint supply framework led by the government and supplemented by enterprises, NGOs and family caregivers. Policy design should incorporate standardized integrated sports-medical-older adults care services into the coverage of rural pension and medical insurance systems, integrate scattered public health resources, and guarantee the physical and mental health of rural older adult groups.

##### 3.1.4.2 Long-term government financial investment guarantee mechanism

Long-term government financial investment is the key to ensuring the efficient and coordinated operation of the three subsystems—sports, healthcare, and care services for older adults—and plays an irreplaceable role in providing basic support. At present, the supply of care services for older adults in rural Shandong shows obvious regional imbalance. To improve service accessibility, provincial and municipal governments need to establish stable long-term financial investment mechanisms, accelerate the construction of rural fitness venues and grassroots medical and care facilities for older adults, and increase the reserve of professional fitness instructors, general practitioners and nursing staff. Optimizing salary security and vocational training systems for grassroots service practitioners can effectively improve the stability and professional capacity of frontline service teams (11). At the same time, we will continue to allocate fiscal funds to the renovation of rural outdoor public spaces, age-friendly home modifications, and the construction of public transportation infrastructure, with the aim of creating age-friendly rural communities and steadily improving the overall quality of life for middle-aged and older people in rural areas (12).

##### 3.1.4.3 Multi-subject joint risk response mechanism

From the perspective of synergy theory, the establishment of this collaborative risk response mechanism is intended to prevent the entire service system from once again falling into a state of disorder and departmental fragmentation. All relevant functional departments and service institutions need to jointly respond to operational risks of rural aging health services. Local governments can set up special coordinated funds to buffer the operational pressure of grassroots care facilities for older adults and provide targeted living subsidies for advanced-age, disabled and low-income rural older adult groups.

Universal publicity of scientific fitness, chronic disease management and standardized knowledge of care services for older adults should be carried out in villages and community service stations to narrow the gap between rural residents’ health cognition and professional service standards, and continuously lift the overall quality of life for rural older adults (13).

### 3.2 Realistic dilemmas of integrated sports-medical-older adults care services in rural Shandong

#### 3.2.1 Overall development lag of rural integrated aging services

The development of integrated sports-medical-older adults care in rural Shandong falls far behind urban areas, with three prominent bottlenecks: First, rural fitness infrastructure and professional fitness guidance resources are severely insufficient. Most rural older adults can only carry out simple walking and outdoor rest activities, lacking scientific, targeted chronic disease rehabilitation exercise plans; self-care disabled older adults face extremely limited activity options, bringing hidden dangers to long-term physical health management. Second, grassroots medical resources are constrained: professional medical staff shortages, outdated diagnosis and treatment equipment and simple service environments make it difficult to meet the daily chronic disease intervention needs of rural older adult patients. Third, grassroots care facilities for older adults in rural areas generally face sustained funding shortages. Most village-level care facilities rely on public finance with outdated hardware and single service content, failing to form differentiated service hierarchies to meet multi-level aging demands (14). Building a unified integrated sports-medical-older adults care system is an essential way to make up for rural public health service weaknesses under the early health intervention strategy.

#### 3.2.2 Inevitable trend of integrated services under deep rural aging

Official data show that Shandong ranks first nationwide in total older adults population, aging ratio and aging burden. By the end of 2025, the population aged 60 and above accounted for 25.70% of the provincial population, and the population aged 65 and above accounted for 18.13% (1). Demographic projections show the provincial aging rate will break 30% by 2035, entering a super-aging society with continuously intensifying pressures on care services for older adults.

Chronic metabolic diseases account for approximately 70% of China’s total disease burden (15), making early preventive fitness intervention a core public health priority. Mass rural labor outflow has impacted the traditional family-based care model for older adults, exposing structural problems such as single service supply structure and low service quality in rural aging systems.

The report to the 19th CPC National Congress clearly proposed actively responding to population aging and promoting medical-older adults care integration. The sports-medical-older adults care integrated model integrates scientific exercise intervention, routine medical treatment and daily aging care, realizes resource sharing across three sectors, effectively prevents chronic disease deterioration and improves the physical and mental health of rural older adults, becoming an inevitable development trend of rural aging governance in Shandong (16).

#### 3.2.3 Supply–demand imbalance restricting integrated service development

##### 3.2.3.1 Insufficient rural public service resources with prominent supply–demand mismatch

Shandong’s rural areas hold a huge older adults population with high chronic disease prevalence, yet standardized integrated service institutions are scarce with outdated supporting facilities. Government financial investment in rural social aging services is inadequate, and service charging standards exceed the bearing capacity of most rural families, leading to low actual service coverage. Most rural older adults still rely solely on family self-care rather than institutional or community integrated services (17).

##### 3.2.3.2 Narrow service coverage and low standardized service quality

Under traditional family and community care modes, rural caregivers are mostly family members or older adult volunteers without professional training, unable to provide standardized chronic disease rehabilitation, scientific fitness guidance and professional nursing services. International aging governance research provides reference experience: even in welfare-developed countries such as Sweden, family informal caregivers remain the main support force for older adult groups, and targeted policy support for family caregivers is a core reform direction of long-term care systems (18). Service satisfaction evaluation mechanisms in European care institutions for older adults also show that service quality, staff professionalism and facility conditions are core factors affecting older adult users’ experience (3). Cross-national research further confirms that socioeconomic status and physical functional impairment are key variables affecting the accessibility of formal care services for older adults, requiring differentiated inclusive supply design (19).

##### 3.2.3.3 Multi-party cost-bearing conflicts and operational risk pressures

Integrated sports-medical-older adults care services have higher operating costs than traditional single-function care models for older adults. Sole reliance on government financial investment cannot sustain large-scale rural service coverage, while most rural families cannot afford full self-payment of integrated service fees. In the initial construction stage of rural integrated service systems, grassroots institutions also face multiple risks including capital chain pressure, talent shortage and cross-department coordination barriers (20).

#### 3.2.4 Unique value of sports intervention in early geriatric health management

A large body of domestic and international public health research verifies that standardized scientific exercise acts as an effective auxiliary intervention for older adults chronic diseases: it improves body balance and motor coordination to reduce fall risks, enhances respiratory and circulatory function, and lowers overall chronic disease incidence, comprehensively improving physical and mental well-being of older adults. A study on physical activity interventions for older adults in rural China indicates that older adults in rural areas generally suffer from insufficient physical activity and excessive sedentary behavior. A multi-level intervention model based on three dimensions—individual, interpersonal, and community—can effectively increase physical activity participation among older adults in rural areas and demonstrates good adaptability to rural settings and practical feasibility (21). The International Expert Consensus System published by the International Council of Sport Science and Physical Education states that personalized, multifaceted exercise interventions are a core strategy worldwide for slowing physiological aging, preventing and controlling chronic diseases, and maintaining physical activity capacity in older adults (4). Sports-based aging care has become a mainstream healthy aging development direction worldwide. The sports-medical-older adults care integration model organically unifies sports-medical fusion, medical-older adults care fusion and fitness-based aging care, forming a complete early prevention service chain adapted to rural Shandong’s development reality.

### 3.3 Policy optimization paths for rural integrated sports-medical-older adults care service systems

Rural sports-medical-older adults care integration in China remains in the exploratory promotion stage. Local governments need to formulate differentiated development plans according to regional economic and population characteristics, and optimize the top-level design of rural aging public service systems. Relying on the medical resources of township health centers, this paper proposes a six-in-one integrated service framework covering fitness guidance, medical diagnosis, daily care services for older adults, professional nursing, rehabilitation training and health preservation management, forming an inclusive rural aging service model matching Shandong’s rural conditions. Cultivating diversified service suppliers and perfecting multi-stakeholder collaborative supply mechanisms are core breakthroughs to lift rural integrated service capacity (22–24).

#### 3.3.1 Improve provincial-rural tiered policy support system and strengthen government leading capacity

Combined with Shandong’s rural aging population characteristics, a complete supporting policy system for integrated sports-medical-older adults care should be constructed at all government levels. Special financial subsidies should be targeted at advanced-age, disabled and low-income rural older adult groups, and the promotion of insurance for care services for older adults in rural areas should be accelerated. The government can purchase public aging services from social professional institutions to expand service supply channels (12, 25, 26).

Special cross-department coordination working groups should be established to break administrative barriers between sports, health and civil affairs departments, and build a unified service supervision and effect evaluation system. Policy support for social welfare organizations should be increased to make up for the shortage of rural integrated service capacity.

#### 3.3.2 Expand the coverage scope of social medical and long-term care insurance

Eligible medical service units within rural care institutions for older adults should be incorporated into designated medical insurance providers, raising the reimbursement proportion of older adult chronic disease diagnosis and rehabilitation treatment. Empirical evidence indicates that medical insurance reimbursement levels are closely correlated with self-rated health outcomes among rural middle-aged and older adults (35). Critical illness insurance coverage should fully cover rural low-income older adult groups with differentiated preferential reimbursement policies.

Local exploration of rural long-term care insurance mechanisms should be encouraged, with qualified township medical and care facilities for older adults designated as insurance service providers. Rehabilitation projects including cognitive function training and hemiplegia limb exercise should be included in basic medical insurance reimbursement scope to provide systematic insurance guarantee for older adult groups with varying degrees of physical dysfunction. While improving financing support, attention should also be paid to optimizing service processes and user experience of community-based care services, as reflected in urban community meal service practice (34).

#### 3.3.3 Build a professional talent training system for integrated sports-medical-older adults care services

Sustainable development of integrated aging services relies on a stable professional talent team covering sports rehabilitation, clinical medical treatment and older adults nursing. Higher vocational colleges and university sports and medical majors should expand relevant training directions, deepen industry-education integration, and deliver professional practitioners matching rural service demands.

Continuous on-the-job training systems should be built for existing grassroots service staff to upgrade their capacity in older adults chronic disease identification, scientific fitness prescription and standardized nursing, providing lasting talent support for rural integrated service construction.

#### 3.3.4 Improve whole-process supervision, assessment, and establish integrated village-level service hubs with a remote health and wellness guidance system

Form standardized whole-process supervision and reward-punishment assessment systems for integrated sports-medical-older adults care services. Special coordination departments should be set up at the county level to promote seamless cooperation among sports, medical, and care facilities for older adults, thereby raising the overall operational efficiency of integrated services.

Develop and implement age-appropriate scientific fitness guidance programs, and gradually establish a network of village-level fitness facilities covering all administrative villages. Establish integrated service centers of medical care and care services for older adults in each administrative village, equip each center with basic videoconferencing equipment, and build a provincial-level remote health and wellness guidance platform. Utilize digital technology to provide rural seniors with professional exercise prescriptions, chronic disease management, and rehabilitation training services. Encourage universities and research institutions to develop smart fitness monitoring and rehabilitation devices for older adults to enhance the scientific rigor and safety of exercise interventions for rural seniors. Conduct widespread public awareness campaigns in rural areas on healthy aging and national policies on care services for older adults to shift public perceptions away from a rigid reliance on traditional family-based care, and guide partially disabled and older adult individuals to professional, integrated health and wellness institutions. Recognize high-quality service providers through standardized evaluation mechanisms to foster a social atmosphere of respect and care for older adults.

For integrated sports, medical care, and care services for older adults in rural areas to operate sustainably, the key lies in the coordinated allocation of differentiated resources and governance innovations tailored to local conditions; simply replicating urban service development models is not feasible. Developing tiered service plans tailored to local needs and leveraging digital tools to empower grassroots services are crucial to improving access to health and wellness services for rural older adults (27–30). Based on the internal mechanisms by which physical activity influences the health of rural older adults through the multiple mediating effects of educational attainment, household income, and psychological capital, Sun (6) proposes the establishment of a targeted, interconnected, and sustainable health security system for rural older adults. Building on this conclusion, this study further expands and refines the theoretical framework: by leveraging village-level comprehensive service stations to establish a remote health and wellness platform and promoting a model that combines in-person group participation with online group physical exercise, the study aims to bridge the digital divide faced by left-behind older adults in rural areas and alleviate the loneliness associated with living alone. This blended online-offline service model can fully harness the positive empowering effects of educational attainment, economic income, and psychological capital on the physical and mental health of rural older adults, ultimately establishing a closed-loop rural health promotion system with physical activity as its core lever. Drawing on the mature practices of remote health and wellness care in Australia’s remote rural areas summarized by O'Connell et al. (31), organize rural seniors to participate synchronously in online group fitness classes through physical service sites. This blended online-offline model not only effectively breaks down the operational barriers that prevent older adults living alone from using digital devices and alleviates the loneliness associated with exercising alone at home, but also fully harnesses the social value of group exercise, yielding dual physical and mental wellness benefits (27, 28, 30, 31).

### 3.4 Discussion

Against the dual backdrop of China’s implementation of early preventive health intervention strategies and the continuing deepening of the aging population in rural Shandong Province, delaying physical aging, reducing the risk of chronic diseases, and achieving equitable and sustainable healthy aging have become core governance priorities for local governments at all levels. Optimizing the allocation of cross-departmental resources and promoting the coordinated development of sports, healthcare, and care services for older adults have also gradually become the mainstream direction for reforming the rural public health system. In this context, establishing an integrated service system that aligns with local realities and is feasible for implementation is a key measure to resolve the structural contradiction between the growing health needs of older adults and the insufficient supply of rural public services.

Adhering to the core concept of shifting health governance from reactive treatment after illness to proactive prevention, this study systematically analyzed the existing endowments, practical challenges, and institutional barriers of rural sports, medical, and care services for older adults resources in Shandong Province. Based on this analysis, it constructed a standardized theoretical framework and operational system for integrated services to address the multifaceted challenges posed by rural population aging. Through cross-sectoral resource integration and collaborative governance among multiple stakeholders, this system can comprehensively improve the physical and mental health of rural older adults, meet their multi-level and diverse health and wellness needs, and effectively enhance the overall quality of healthy aging in rural areas.

The findings of this study align with existing domestic and international research: fragmented departmental governance, uneven resource allocation, and a shortage of professional service provision are universal challenges constraining improvements in the quality of health services for rural older adults (3, 18, 20, 32). Numerous previous empirical studies have also confirmed that scientifically grounded exercise interventions play an irreplaceable role in the prevention and control of chronic diseases among older adults and in maintaining physical function, which further validates the core value of integrating fitness guidance into care services for older adults in rural areas (8, 17). It is important to distinguish between generalizable findings and Shandong-specific observations from this analysis. The core challenges including cross-sector fragmentation, shortage of professional workforce, and unmet demand for proactive early health interventions can be generalized to many aging rural regions across China and other middle-income countries with rural population outflow. However, several characteristics are uniquely rooted in Shandong’s context: its large absolute size of rural older adults population, provincial policy priorities for sports and public health, and the provincial administrative structure that shapes cross-department coordination between sports, health and civil affairs bureaus. These contextual features mean that the detailed operational mechanisms and policy pathways proposed here cannot be directly copied into other provinces without adjustment to match local fiscal capacity, demographic structure and institutional arrangements.

Nevertheless, this study makes innovative contributions to the existing research framework across three key dimensions. First, most existing studies focus on urban community settings, emphasizing post-illness rehabilitation and passive care services for older adults, with very few oriented toward early preventive health interventions or aimed at establishing a systematic service framework for rural older adults; this study shifts the research focus from end-of-life care to proactive health management across the entire life cycle. Second, previous macro-level discussions at the national level have failed to incorporate the typical characteristics of rural aging in Shandong and the current state of grassroots governance to develop localized, tailored designs. This study fully considers regional resource constraints and the actual health and care needs of rural older adults, resulting in a system that is more targeted and practical. Third, most previous studies have only offered scattered policy recommendations and have failed to establish a comprehensive, multi-stakeholder collaborative mechanism; this study clarifies the logic of collaboration among various stakeholders—including government departments, primary healthcare institutions, villages and communities, and families—effectively filling this research gap.

At the theoretical level, this conceptual study further enriches the connotations of the “Healthy China” strategy, the concept of “comprehensive health across all regions,” and the theory of full-life-cycle health management; it expands the localized application of multi-stakeholder collaborative governance theory in the integration of rural public health and care services for older adults, and can provide theoretical material and teaching references for academic research in the fields of sports public health and rural social governance (33). At the practical level, the optimized “six-in-one” service model and its supporting governance pathways can provide actionable policy guidance for local governments to address the challenges of rural aging, coordinate and allocate cross-departmental resources, and enhance the equity and sustainability of public health services for rural older adults.

This study has several limitations: First, the analysis is based solely on a review of the literature and publicly available official statistics, and does not include empirical validation through field research, questionnaire surveys, or quantitative assessments. Future research could conduct field studies to examine the actual implementation of the service system proposed in this paper and to evaluate service satisfaction among older adult users. Second, this study focuses on overall service provision and general rural governance needs; it does not distinguish regional development disparities within Shandong Province, nor does it categorize the stratified health needs of different older adults populations. Future research could employ stratified sampling and cross-regional comparative analysis to establish a differentiated service delivery mechanism. Third, while this study provides a preliminary exploration of how digitalization and intelligent technologies can enhance health and wellness services, it does not systematically integrate smart health monitoring devices into the overall service framework. Incorporating smart sensing and monitoring devices when optimizing the system architecture in the future could effectively improve the accuracy and scientific rigor of early preventive health interventions in rural care settings for older adults in rural areas.

## 4 Conclusion

From the perspective of early preventive health interventions and supported by a localized adaptation of the theory of synergy, this paper employs a two-stage systematic literature review method to systematically examine the theoretical framework, current status, and practical challenges of the integrated service system of physical fitness, medical care, and care services for older adults in rural Shandong Province, and draws key research conclusions. Currently, rural areas in Shandong generally suffer from weaknesses such as inadequate fitness facilities, insufficient primary healthcare resources, a shortage of professional personnel, and limited fiscal investment. Meanwhile, the multi-level and diverse health needs of the older adults population continue to grow, resulting in a prominent structural mismatch between the supply and demand for public services.

Unlike the collaborative health and care service systems for older adults in Western countries—which rely on mature social organizations and market-based mechanisms—and distinct from the small-scale, high-density, community-led health service models for older adults in Japan and South Korea, the integrated development of fitness, medical care, and care services for older adults in China’s rural areas is highly dependent on coordinated government governance. At the same time, it faces unique governance challenges such as departmental fragmentation, scattered resources, and weak grassroots capacity. This study, taking into account the characteristics of rural governance in China, has established five core operational mechanisms: collaborative support from multiple stakeholders, long-term fiscal investment, universal social security coverage, professional talent development, and comprehensive risk supervision throughout the entire process. It also proposes targeted policy optimization pathways that can effectively optimize the allocation of health resources for rural older adults and promote the standardized and sustainable development of integrated physical fitness, medical care, and care services for older adults.

The findings of this study not only provide a theoretical basis and practical reference for Shandong Province to improve its public service system of care services for older adults in rural areas and deepen the development of healthy aging, but also offer a localized and adaptable practical paradigm for developing countries in East Asia, Southeast Asia, and elsewhere that share characteristics of government-led governance models and rapid rural aging. Furthermore, they contribute Chinese rural experience to the international academic community’s efforts to refine theoretical frameworks on active aging and the collaborative governance of public services. Future follow-up studies can advance this work in multiple directions. First, cross-provincial comparative research can test the transferability of this synergy-based service framework in rural regions with different economic and demographic profiles, helping separate universal governance rules from region-specific policy adaptations. Second, empirical field investigations and questionnaire surveys can be implemented in Shandong’s rural villages to collect stakeholder and end-user data, quantify service accessibility and satisfaction, and empirically validate the conceptual framework constructed in this paper. Third, future research may develop quantitative indicators to measure synergy degrees across sports, medical and care services for older adults subsystems, enabling dynamic evaluation of system performance. In addition, stratified analysis within Shandong can compare eastern and western rural counties to capture intra-provincial disparities and design differentiated tiered service schemes.
